# Tyrosine-kinase inhibitor combined with iodine-125 seed brachytherapy for hepatocellular carcinoma refractory to transarterial chemoembolization: a propensity-matched study

**DOI:** 10.1186/s40644-023-00604-4

**Published:** 2023-09-25

**Authors:** Yongjian Guo, Jingqiang Wu, Licong Liang, Kangshun Zhu, Jingwen Zhou, Liteng Lin, Ye Chen, Bihui Cao, Mingji He, Hui Lian, Wensou Huang, Mingyue Cai

**Affiliations:** 1 Department of Minimally Invasive Interventional Radiology, The Second Affiliated Hospital of Guangzhou Medical University, Guangzhou, Guangdong China; 2 Radiology Center, The Second Affiliated Hospital of Guangzhou Medical University, Guangzhou, Guangdong China

**Keywords:** Hepatocellular carcinoma, Therapeutic chemoembolization, Tyrosine kinase inhibitor, Brachytherapy, Combined modality therapy

## Abstract

**Purpose:**

To investigate the efficacy and safety of tyrosine-kinase inhibitor (TKI) combined with iodine-125 seed brachytherapy (TKI-I) versus TKI alone for patients with hepatocellular carcinoma (HCC) refractory to transarterial chemoembolization (TACE).

**Methods:**

Data of patients with TACE-refractory HCC who received TKI (sorafenib or lenvatinib) or TKI-I from September 2018 to December 2020 were retrospectively analyzed. A propensity score matching (PSM) was performed to diminish potential bias. The primary endpoints were overall survival (OS) and time to progression (TTP). Tumor responses and treatment-related adverse events (TRAEs) were also compared between the two groups.

**Results:**

A total of 132 patients were included in this study. Under PSM, 48 paired patients were selected for comparison. The median OS was 23.2 (95% CI 20.9–25.1) months in the TKI-I group versus 13.9 (95% CI 11.1–16.7) months in the TKI group (*P* < 0.001). The median TTP was 12.8 (95% CI 10.1–15.5) months in the TKI-I group versus 5.8 (95% CI 5.0-6.6) months in the TKI group (*P* < 0.001). Patients in the TKI-I group had higher objective response rate (68.8% vs. 33.3%, *P* = 0.001) and disease control rate (89.6% vs. 66.7%, *P* = 0.007) than those in the TKI group. The incidence and severity of TRAEs in the TKI-I group were comparable to those in the TKI group (any grade, 89.7% vs. 92.2%, *P* = 0.620; ≥grade 3, 33.8% vs. 32.8%, *P* = 0.902).

**Conclusions:**

TKI-I was safe and significantly improved survival over TKI alone in HCC patients with TACE refractoriness.

**Supplementary Information:**

The online version contains supplementary material available at 10.1186/s40644-023-00604-4.

## Introduction

Hepatocellular carcinoma (HCC) is the most common type of primary liver cancer, which is the third leading cause of cancer-related death worldwide [1]. Although disease at early stage may be curable by ablation, surgical resection or liver transplantation, the majority of patients are diagnosed with unresectable disease and thus have a poor prognosis [2–4]. Transarterial chemoembolization (TACE) is one of the most widely used nonsurgical treatments for unresectable HCC [2, 3]. In clinical practice, repeated TACE is often performed for maximizing treatment outcomes. However, it may lose its efficacy at some point, and in turn lead to deterioration of liver function and even worse, have adverse effects on survival [5–8]. On this condition, repetition of TACE is no longer beneficial and the patients enter the state termed TACE failure/refractoriness. It is recommended that, once TACE refractoriness occurs, the patients should be switched to other treatment modality immediately [5, 6].

Since randomized trials have demonstrated improved survival with sorafenib versus placebo [9, 10] and noninferiority of lenvatinib to sorafenib [11], these two tyrosine-kinase inhibitors (TKIs) have been recommended as the first-line treatment options for advanced HCC [2, 3, 12]. Some studies [13, 14] have suggested that patients who switched to sorafenib after TACE refractoriness had a better prognosis than those continually treated with TACE. However, considering the persistently residual or progressive tumor on repeated TACE [5, 6] and the limited tumor response and survival prolongation with oral sorafenib [15, 16], the therapeutic outcomes of TKI monotherapy for TACE-refractory HCC may not be satisfactory, which brings forth an urgent demand for more effective treatment strategies.

Radiotherapy is a well-known local-regional treatment for many types of cancers, including HCC [17]. Iodine-125 seeds are a radioactive source for brachytherapy which can be implanted into the tumor and generate an even and quantifiable radiation dose distribution within the target area, while causing little radiation toxicity to surrounding normal tissues [18, 19]. Previous studies, including ours, have revealed that iodine-125 seed brachytherapy alone or plus other treatments was effective for HCC, with a sufficient tumor control as well as an increased survival [19–24]. As radiotherapy can improve tumor response in HCC patients treated with sorafenib or lenvatinib and these TKIs can enhance sensitivity of tumors to radiation [25–29], combining TKI (sorafenib or lenvatinib) with iodine-125 seed brachytherapy (TKI-I) may possess a better anticancer activity than TKI alone on HCC refractory to TACE. Therefore, we conducted this retrospective study to investigate the efficacy and safety of TKI-I compared with TKI alone in HCC patients with TACE refractoriness.

## Methods

### Study design and patient selection

This study was approved by the Ethics Committee of the Second Affiliated Hospital of Guangzhou Medical University (approval number, 2022-KY-ks-05), and the requirement of informed consent was waived. Data of consecutive HCC patients with TACE refractoriness who underwent TKI-I (TKI-I group) or TKI (TKI group) treatment at our institution from September 2018 to December 2020 were retrospectively analyzed.

The inclusion criteria were as follows: (1) age from 18 to 75 years; (2) diagnosis of HCC with TACE refractoriness according to the criteria proposed by Japan Society of Hepatology [30]; (3) Eastern Cooperative Oncology Group performance status (ECOG PS) score ≤ 1; and (4) Child-Pugh class A/B liver function. The exclusion criteria were: (1) incomplete medical records; (2) extrahepatic spread; (3) tumor thrombus involving the main portal vein or vena cava; (4) previous treatment with hepatic arterial infusion chemotherapy, radiotherapy or systemic therapy; (5) history of malignancies other than HCC; (6) history of organ transplantation; (7) prolongation of prothrombin time ≥ 4 s or platelet count < 50 × 10^9^/L; (8) severe cardiac, pulmonary or renal dysfunction.

All baseline laboratory test and computed tomography (CT) or magnetic resonance imaging (MRI) data were collected within one week before the initiation of TKI-I or TKI treatment.

### TKI administration

All patients received sorafenib (Bayer Pharma, Leverkusen, Germany) or lenvatinib (Eisai, Tokyo, Japan) after TACE refractoriness was determined. Sorafenib at a dose of 400 mg was administered orally twice a day. Lenvatinib at a dose of 12 mg (bodyweight ≥ 60 kg) or 8 mg (bodyweight < 60 kg) was administered orally once a day. Interruption and dose reduction of TKI was allowed and depended on the presence and severity of toxicities according to the package insert. TKI treatment was continued until intolerable toxicity or disease progression occurred.

### Iodine-125 seed implantation

Iodine-125 seed brachytherapy was indicated for patients with non-diffuse viable intrahepatic tumor and/or vascular tumor thrombosis who had good performance status (ECOG PS ≤ 1), Child-Pugh class A/B liver function, prothrombin time prolongation < 4 s and platelet count ≥ 50 × 10^9^/L. Whether to combine iodine-125 seed brachytherapy or not was determined according to the physicians’ recommendation after discussion and the patients’ choice. For the patients treated with TKI-I, CT-guided iodine-125 seed implantation was performed within a week before or after TKI administration.

The iodine-125 seed (ZHIBO Bio-Medical Technology, Beijing, China) was shaped as cylinder with the following parameters: diameter of 0.8 mm; length of 4.5 mm; radioactivity of 0.6–0.8 mCi; initial dose rate of 8–10 cGy/h; energies of 27.4, 31.4 KeV for X-ray and 35.5 KeV for γ-ray; radioactive half-life of 60.1 days; and tissue half-value layer of 1.7 cm. Before seed implantation, abdominal CT/MRI images with 5-mm slice thickness were transmitted to a seed brachytherapy treatment planning system (Tianhang Kelin Technology Development, Beijing, China) and a preoperative planning was developed to determine the number and locations of seeds according to a prescription dose of 110–160 Gy, which allowed a complete coverage of the viable intrahepatic tumor and/or tumor thrombus. Iodine-125 seeds were implanted into the target lesions under CT guidance with 18-gauge Chiba needles and implant guns containing the seeds in the cartridge chamber. The space interval between adjacent seeds was 0.5-1.0 cm. Immediately after seed implantation, a CT scan was re-performed for verifying the distribution of iodine-125 seeds and assessing whether bleeding or other complications occurred.

If patients were found to have insufficient radioactive coverage of tumors by follow-up imaging, iodine-125 seed implantation was repeated based on a consensus after discussion by the attending physicians. The repeated procedure was performed only when the patients had a ECOG PS ≤ 2 with Child-Pugh class A/B, prothrombin time prolongation < 4 s and platelet count ≥ 50 × 10^9^/L.

### Follow-up

The patients were followed up at an interval of 4–8 weeks until death or their last follow-up. Each follow-up session included a detail history, physical examination, laboratory tests, contrast-enhanced abdominal CT or MRI, chest CT and other imaging examination if clinically indicated. The final follow-up ended on December 31, 2021.

### Assessments and outcomes

The primary endpoints of this study were overall survival (OS) and time to progression (TTP). OS was defined as the time interval from diagnosis of TACE refractoriness to the time of death from any reason. TTP was defined as the time interval from diagnosis of TACE refractoriness to the first occurrence of disease progression.

Tumor response was classified into complete response (CR), partial response (PR), stable disease (SD) or progressive disease (PD) according to modified Response Evaluation Criteria in Solid Tumors [31]. For patients with vascular invasion, treatment response of tumor thrombus was evaluated by using a modified standard: CR, complete disappearance or shrinkage of thrombus, or complete disappearance of enhancement inside thrombus; PR, ≥ 30% decrease in the largest perpendicular diameter of thrombus, or thrombus shrinking back to a higher-order branch of portal vein; PD, ≥ 20% increase in the largest perpendicular diameter of thrombus, or thrombus extending to a more proximal portal vein; SD, a tumor thrombus response between PR and PD. Objective response rate (ORR) was defined as the percentage of patients with a best tumor response rating of CR and PR. Disease control rate (DCR) was defined as the percentage of patients with a best tumor response rating of CR, PR and SD.

Adverse events (AEs) were recorded and evaluated in accordance with Common Terminology Criteria for Adverse Events version 5.0. Treatment-related AEs (TRAEs) were monitored until 90 days after the discontinuation of TKI-I or TKI treatment.

### Statistical analyses

A 1:1 propensity score matching (PSM) analysis was performed to minimize the potential selection bias. The propensity score was calculated by a logistic regression model using a caliper of 0.02 with variables of age, ECOG PS (0/1), Child-Pugh class (A/B), α-fetoprotein (AFP), number of tumors, largest tumor size, tumor distribution (bilobar/unilobar) and macrovascular invasion (yea/no) [32, 33]. Categorical data were presented as number of patients (percentage) and were compared by using χ2 test. Quantitative data (non-normally distributed) were presented as median (range) and were compared by using Mann-Whitney U test. OS and TTP curves were generated by Kaplan-Meier method and compared by using log-rank test. Univariate and multivariate analyses of prognostic factors for OS and TTP were conducted using Cox proportional hazard regression model. Variables with *P* < 0.10 in the univariate analysis were entered into the multivariate analysis. All statistical analyses were performed with SPSS Statistics, version 26 (IBM, Armonk, New York, USA). All tests were two-tailed, *P* < 0.05 was considered statistically significant.

## Results

### Study population

**Fig. 1:**
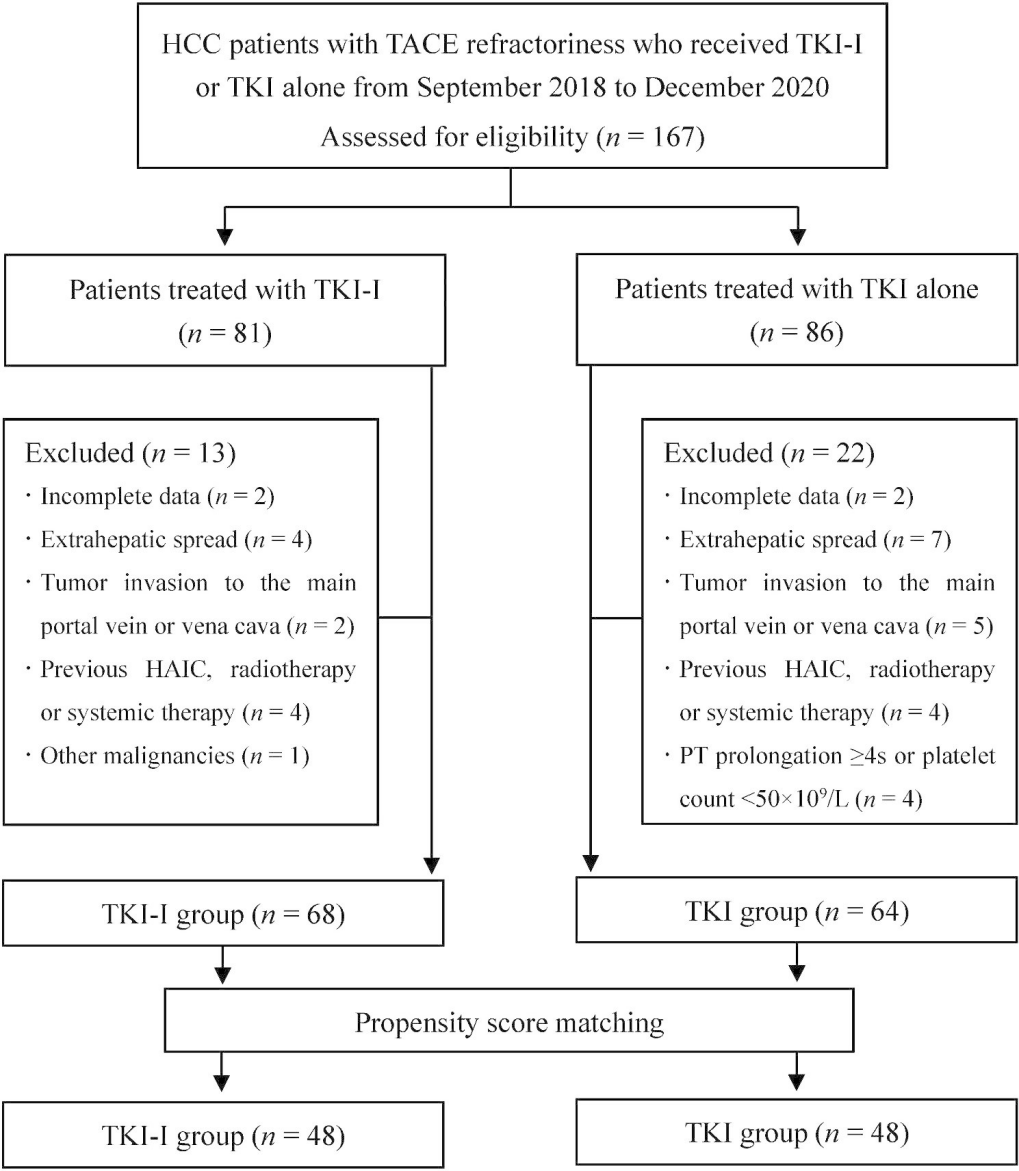
Flowchart of patient selection. *HCC* hepatocellular carcinoma, *TACE* transarterial chemoembolization, *TKI-I* tyrosine-kinase inhibitor combined with iodine-125 seed brachytherapy, *TKI* tyrosine-kinase inhibitor, *HAIC* hepatic arterial infusion chemotherapy, *PT* prothrombin time

During the study period, a total of 167 HCC patients with TACE refractoriness were treated with TKI-I or TKI alone at our hospital. Of these patients, 35 were excluded in that they met the excluded criteria. As a result, 132 patients were included in this study: 68 in the TKI-I group and 64 in the TKI group (Fig. 1). Before PSM, there were more patients had Child-Pugh class A liver function (*P* = 0.030), tumor number ≤ 3 (*P* = 0.023) or unilobar tumor distribution (*P* = 0.012) in the TKI-I group. Following PSM, 48-paired patients with well-balanced baseline characteristics were further selected (Table 1).

**Table 1:** Baseline characteristics of the patients

Characteristic	Total cohort				Matched cohort		
	TKI-I group (*n* = 68)	TKI group (*n* = 64)	*P*		TKI-I group (*n* = 48)	TKI group (*n* = 48)	*P*
Sex							
Male	58 (85.3)	57 (89.1)	0.518		40 (83.3)	44 (91.7)	0.217
Female	10 (14.7)	7 (10.9)			8 (16.7)	4 (8.3)	
Age (years)	55.0 (29.0–75.0)	58.5 (28.0–75.0)	0.398		57.5 (35.0–75.0)	60.0 (28.0–75.0)	0.538
< 60	42 (61.8)	32 (50.0)	0.173		25 (52.1)	23 (47.9)	0.683
≥ 60	26 (38.2)	32 (50.0)			23 (47.9)	25 (52.1)	
ECOG PS							
1	22 (32.4)	27 (42.2)	0.242		15 (31.3)	15 (31.3)	> 0.999
0	46 (67.6)	37 (57.8)			33 (68.8)	33 (68.8)	
HBsAg							
Positive	56 (82.4)	58 (90.6)	0.166		40 (83.3)	44 (91.7)	0.217
Negative	12 (17.6)	6 (9.4)			8 (16.7)	4 (8.3)	
Child-Pugh class							
B	5 (7.4)	13 (20.3)	0.030		5 (10.4)	3 (6.3)	0.712
A	63 (92.6)	51 (79.7)			43 (89.6)	45 (93.8)	
AFP (µg/L)	209.1 (1.5-172903.5)	201.7 (1.3-800000.0)	0.623		276.2 (1.8-172903.5)	167.9 (1.3-800000.0)	0.758
≥ 200	35 (51.5)	32 (50.0)	0.866		27 (56.3)	23 (47.9)	0.414
< 200	33 (48.5)	32 (50.0)			21 (43.8)	25 (52.1)	
Number of tumors^*^	2 (1–10)	3 (1–10)	0.017		2 (1–10)	3 (1–10)	0.129
> 3	17 (25.0)	28 (43.8)	0.023		14 (29.2)	21 (43.8)	0.138
≤ 3	51 (75.0)	36 (56.3)			34 (70.8)	27 (56.3)	
Tumor distribution							
Bilobar	31 (45.6)	43 (67.2)	0.012		25 (52.1)	30 (62.5)	0.302
Unilobar	37 (54.4)	21 (32.8)			23 (47.9)	18 (37.5)	
Largest tumor size (cm)	7.9 (3.0-20.2)	8.2 (3.0-19.8)	0.340		8.3 (3.0-20.2)	8.9 (3.0-19.1)	0.373
> 7.0	42 (61.8)	39 (60.9)	0.922		30 (62.5)	32 (66.7)	0.670
≤ 7.0	26 (38.2)	25 (39.1)			18 (37.5)	16 (33.3)	
Macrovascular invasion							
Yes	55 (80.9)	48 (75.0)	0.415		36 (75.0)	40 (83.3)	0.315
No	13 (19.1)	16 (25.0)			12 (25.0)	8 (16.7)	
TKI							
Sorafenib	29 (42.6)	26 (40.6)	0.814		22 (45.8)	22 (45.8)	> 0.999
Lenvatinib	39 (57.4)	38 (59.4)			26 (54.2)	26 (54.2)	
Number of previous TACE	3 (2–11)	3 (2–9)	0.519		3 (2–7)	3 (2–9)	0.817
2	23 (33.8)	26 (40.6)	0.419		16 (33.3)	20 (41.7)	0.399
> 2	45 (66.2)	38 (59.4)			32 (66.7)	28 (58.3)	
TACE technique							
D-TACE	28 (41.2)	28 (43.8)	0.765		22 (45.8)	24 (50.0)	0.683
cTACE	40 (58.8)	36 (56.3)			26 (54.2)	24 (50.0)	

Data were presented as *n* (%) or median (range). ^*^Six and 7 patients in TKI-I group and TKI group, respectively, in the total cohort, and 4 patients each in TKI-I group and TKI group in the matched cohort had more than 10 intrahepatic tumors, and the number of tumors was counted as 10. *PSM* propensity score matching, *TKI-I* tyrosine kinase inhibitor combined with iodine-125 seed brachytherapy, *TKI* tyrosine kinase inhibitor, *ECOG PS* Eastern Cooperative Oncology Group Performance Status, *HBsAg* hepatitis B surface antigen, *AFP* α-fetoprotein, *TACE* transarterial chemoembolization, *D-TACE* drug-eluting bead transarterial chemoembolization, *cTACE* conventional transarterial chemoembolization

In the matched cohort, patients in TKI-I group had previously undergone a total of 165 TACE procedures with a median of 3 (range, 2–7) per patient, while those in TKI group had undergone a total of 170 TACE procedures with a median of 3 (range, 2–9) per patient. In both groups, 54.2% of the patients received TKI treatment with lenvatinib. The median largest tumor diameter was 8.3 (range, 3.0-20.2) cm and 8.9 (range, 3.0-19.1) cm in the TKI-I group and TKI group, respectively. 75.0% and 83.3% of the patients in TKI-I group and TKI group, respectively, had macrovascular invasion. The median follow-up for the patients was 15.9 (range, 3.2–40.0) months. The median duration of TKI administration was 12.1 (range, 1.4–39.1) months in TKI-I group and 5.4 (range, 0.9–17.2) months in TKI group (*P* < 0.001). A total of 143 iodine-125 seed implantation procedures (median of 3 per patient, range 1–6) were performed for the 48 patients in TKI-I group. The total number of implanted seeds was 4374, with a median of 81.5 (range, 19–186) per patient.

### Overall survival

In the total cohort, 41 patients (60.3%) in TKI-I group and 49 patients (76.6%) in TKI group died during follow-up. The median OS was 21.9 (95% confidence interval [CI] 19.7–24.1) months for TKI-I group and 12.1 (95% CI 8.9–15.3) months for TKI group (*P* < 0.001).

**Fig. 2:**
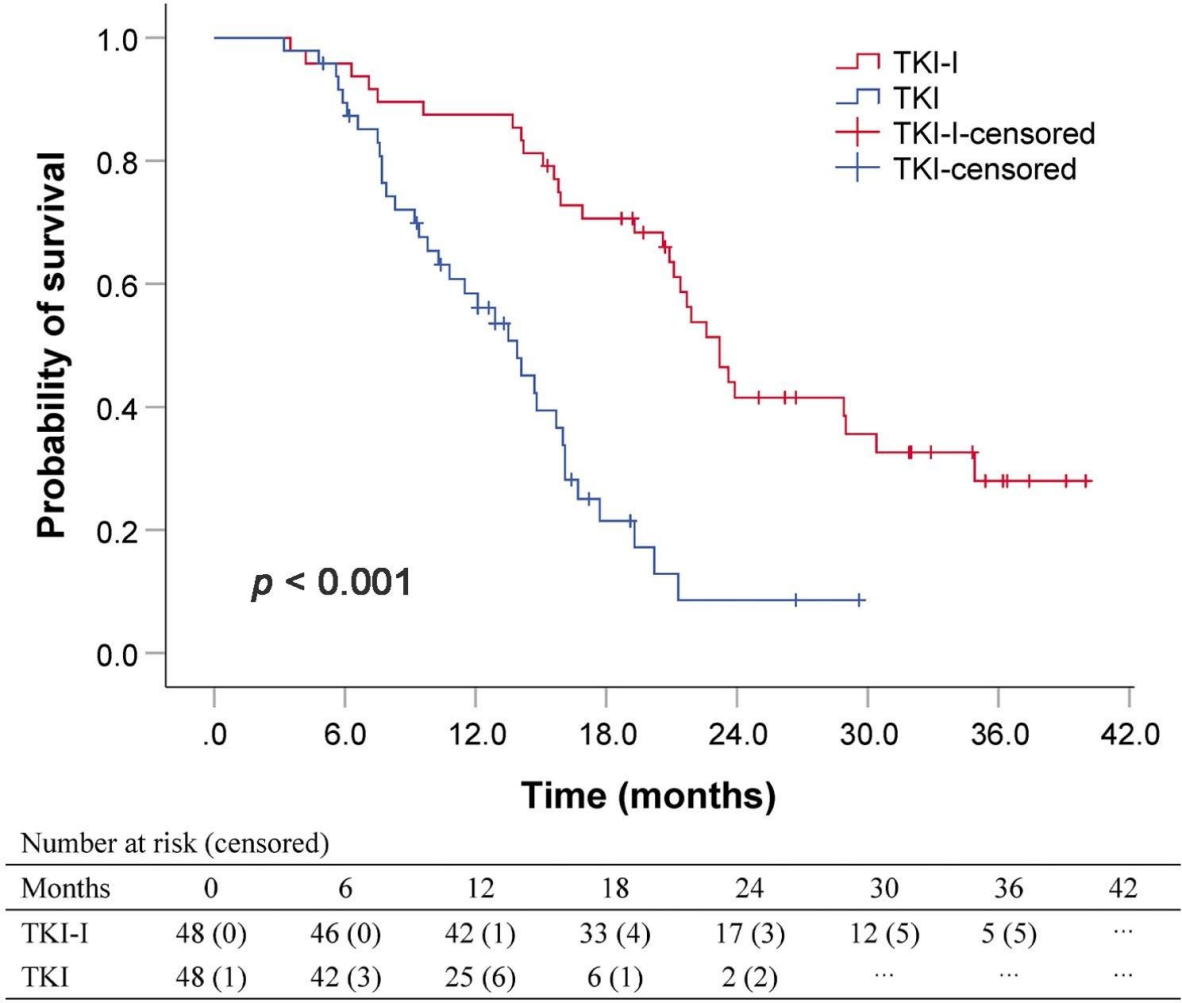
Kaplan-Meier curves for overall survival in the matched cohort according to treatment modality. *TKI-I* tyrosine-kinase inhibitor combined with iodine-125 seed brachytherapy, *TKI* tyrosine-kinase inhibitor

In the matched cohort, 30 patients (62.5%) in TKI-I group and 35 patients (72.9%) in TKI group died during follow-up. The median OS was 23.2 (95% CI 20.9–25.1) months for TKI-I group and 13.9 (95% CI 11.1–16.7) months for TKI group (*P* < 0.001; Fig. 2). Multivariate analysis identified that treatment with TKI alone was an independent adverse prognostic factor for OS (hazard ratio [HR] = 3.546, 95% CI 2.015–6.239, *P* < 0.001; Table S1). Subgroup analyses for OS based on different variables showed that a trend of lower risk of death was achieved with the therapy of TKI-I over TKI alone in almost all the subgroups (Fig. S1).

### Time to progression

In the total cohort, 57 patients in both TKI-I group (83.8%) and TKI group (89.1%) experienced disease progression during follow-up. The median TTP of overall tumor, intrahepatic tumor and vascular tumor thrombus was 11.9 (95% CI 9.2–14.6), 11.9 (95% CI 9.5–14.3) and 15.8 (95% CI 11.2–20.4) months, respectively, for TKI-I group, and 5.7 (95% CI 4.4-7.0), 5.8 (95% CI 4.7-7.0) and 5.9 (95% CI 5.2–6.6) months, respectively, for TKI group (all *P* < 0.001).

**Fig. 3:**
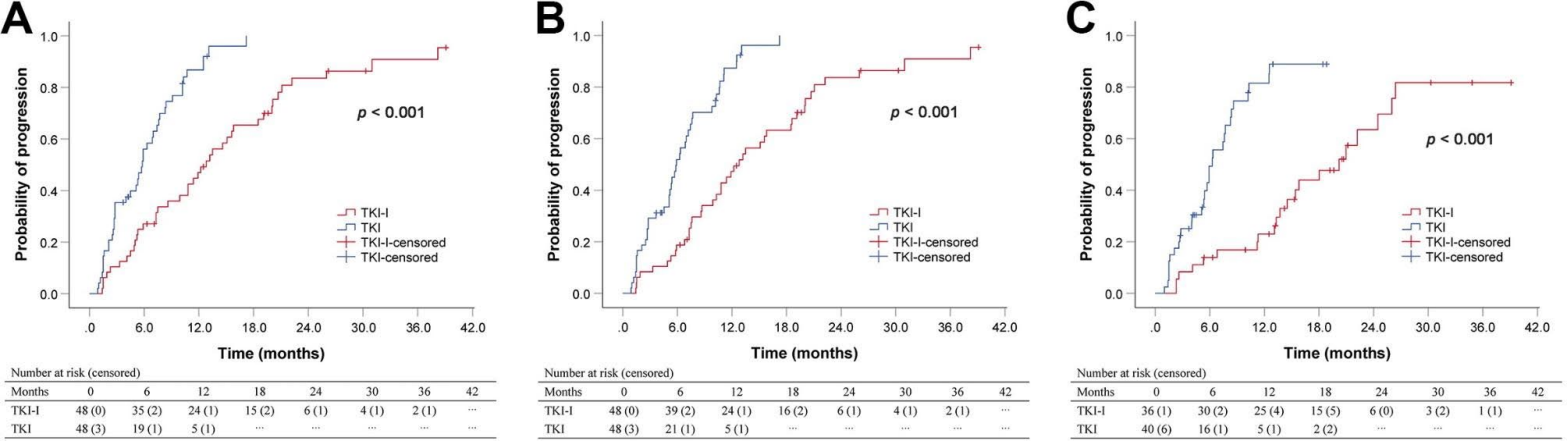
Kaplan-Meier curves for time to progression of (**A**) overall tumor, (**B**) intrahepatic tumor and (**C**) vascular tumor thrombus in the matched cohort according to treatment modality. *TKI-I* tyrosine-kinase inhibitor combined with iodine-125 seed brachytherapy, *TKI* tyrosine-kinase inhibitor

In the matched cohort, 40 patients (83.3%) in TKI-I group and 43 patients (89.6%) in TKI group experienced disease progression during follow-up. The median TTP of overall tumor, intrahepatic tumor and vascular tumor thrombus was 12.8 (95% CI 10.1–15.5), 12.8 (95% CI 9.9–15.7) and 20.2 (95% CI 13.1–27.3) months, respectively, for TKI-I group, and 5.8 (95% CI 5.0-6.6), 5.9 (95% CI 4.7–7.1) and 6.3 (95% CI 5.4–7.1) months, respectively, for TKI group (all *P* < 0.001; Fig. 3A-C). Multivariate analysis identified that treatment with TKI alone was an independent adverse prognostic factor for TTP of overall tumor (HR = 3.305, 95% CI 1.965–5.558, *P* < 0.001; Table S1).

### Tumor responses

In the total cohort, the ORR and DCR of overall tumor (ORR, 61.8% vs. 28.1%, *P* < 0.001; DCR, 88.2% vs. 65.6%, *P* = 0.002), intrahepatic tumor (ORR, 63.2% vs. 29.7%, *P* < 0.001; DCR, 91.2% vs. 68.8%, *P* = 0.001) and vascular tumor thrombus (ORR, 74.5% vs. 12.5%, *P* < 0.001; DCR, 92.7% vs. 64.6%, *P* < 0.001) for TKI-I group were higher than those for TKI group (Table S2).

In the matched cohort, the ORR and DCR of overall tumor (ORR, 68.8% vs. 33.3%, *P* = 0.001; DCR, 89.6% vs. 66.7%, *P* = 0.007), intrahepatic tumor (ORR, 68.8% vs. 35.4%, *P* = 0.001; DCR, 91.7% vs. 70.8%, *P* = 0.009) and vascular tumor thrombus (ORR, 80.6% vs. 12.5%, *P* < 0.001; DCR, 91.7% vs. 67.5%, *P* = 0.010) for TKI-I group were also higher than those for TKI group (Table 2).

**Table 2:** Tumor responses for the patients in matched cohort

Response	Overall tumor				Intrahepatic tumor				Vascular tumor thrombus		
	TKI-I group (*n* = 51)	TKI group (*n* = 51)	*P*		TKI-I group (*n* = 51)	TKI group (*n* = 51)	*P*		TKI-I group (*n* = 42)	TKI group (*n* = 36)	*P*
CR, *n* (%)	1 (2.1)	1 (2.1)			2 (4.2)	1 (2.1)			13 (36.1)	0 (0.0)	
PR, *n* (%)	32 (66.7)	15 (31.3)			31 (64.6)	16 (33.3)			16 (44.4)	5 (12.5)	
SD, *n* (%)	10 (20.8)	16 (33.3)			11 (22.9)	17 (35.4)			4 (11.1)	22 (55.0)	
PD, *n* (%)	5 (10.4)	16 (33.3)			4 (8.3)	14 (29.2)			3 (8.3)	13 (32.5)	
ORR, %	68.8	33.3	0.001		68.8	35.4	0.001		80.6	12.5	< 0.001
DCR, %	89.6	66.7	0.007		91.7	70.8	0.009		91.7	67.5	0.010

*TKI-I* tyrosine kinase inhibitor combined with iodine-125 seed brachytherapy, *TKI* tyrosine kinase inhibitor, *CR* complete response, *PR* partial response, *SD* stable disease, *PD* progressive disease, *ORR* objective response rate, *DCR* disease control rate

### Safety

There was no treatment-related death in the total cohort. The frequency and severity of TRAEs were similar between TKI-I group and TKI group (any grade, 89.7% vs. 92.2%, *P* = 0.620; ≥grade 3, 33.8% vs. 32.8%, *P* = 0.902; Table S3). AEs led to treatment interruption, dose reduction and treatment discontinuation of TKI in 31 (45.6%), 35 (51.5%) and 5 (7.4%) patients, respectively, in TKI-I group, and in 30 (46.9%), 31 (48.4%) and 5 (7.8%) patients, respectively, in TKI group. In TKI-I group, AEs related to iodine-125 seed implantation were observed in 10 patients (14.7%). Among them, right hemothorax (grade 4; successfully managed by intercostal artery embolization) and needle track tumor seeding (grade 3) occurred in one patient each (1.5%).

## Discussion

Our study showed that the treatment with TKI-I was associated with better OS, TTP and tumor response than TKI alone in HCC patients with TACE refractoriness. These fndings were consistently substantiated by the total cohort and the propensity score-matched cohort. Additionally, the frequency of TRAEs in the TKI-I group was similar to that in the TKI group. All these results suggeted that, compared with TKI alone, the addition of iodine-125 seed brachytherapy to TKI might be a superior treatment option for TACE-refractory HCC.

Given that only a marginal survival benefit can be achieved with TKI monotherapy [9–11], sorafenib or lenvatinib has been often combined with other therapies to ameliorate prognosis in HCC patients [16, 34–36]. Previous studies [25–29] have suggested that radiotherapy could enhance treatment response for target lesions in HCC patients treated with sorafenib or lenvatinib. Meanwhile, these TKIs could increase radiosensitivity of tumors. Therefore, combining sorafenib/lenvatinib with iodine-125 seed brachytherapy might elicit synergistic antitumor effects on TACE-refractory HCC. In our study, the ORR, DCR and TTP of overall tumor, intrahepatic tumor and vascular tumor thrombus for TKI-I group were all much better than those for TKI group. We believed that it was the combination of TKI-I that provided a sustained tumor control, thus contributing to the prolonged OS in patients with TACE-refractory HCC.

Previous studies [13, 14, 37] have reported that HCC patients who received sorafenib after TACE refractoriness had a median OS of 20.5–25.4 months, which seemed longer than that for the patients treated with TKI alone in our study. However, it was noteworthy that these studies only enrolled patients with intermediate-stage disease, who were expected to obtain better outcomes than those included in our study (most of the patients had advanced-stage disease with macrovascular invasion). Additionally, the heavy tumor burden the patients borne in our study might also lead to the limited survival benefit. But in any case, compared with TKI alone, TKI-I did provide a significantly improved survival for TACE-refractory patients.

Currently, the treatment of HCC with vascular invasion is still a great challenge [6, 38]. Our study found that TKI-I conferred a higher treatment response of tumor thrombosis compared with TKI alone. More interestingly, with the therapy of TKI-I, the ORR and TTP of tumor thrombosis were better than those of intrahepatic tumors. This might be explained by whether a sufficient radioactive coverage was achieved for the viable tumors [29, 39]. Tumor thrombi were generally confined to the invaded vessels, and their tumor burden was mostly smaller than that of intrahepatic tumors, making them easier to be completely covered by the implanted iodine-125 seeds. Together, these results indicated that iodine-125 seed combination therapy possessed a remarkable therapeutic effect on controlling tumor thrombosis. Accordingly, for TACE-refractory HCC patients with vascular invasion, a more aggressive and effective therapy such as TKI-I was undoubtedly required.

In our study, the incidence and severity of TRAEs in TKI-I group were comparable to those in TKI group. This implied that the addition of iodine-125 seed brachytherapy did not significantly increase the risk of TRAEs in patients treated with TKI. Iodine-125 irradiation has a short penetration radius of 1.7 cm and thus avoid liver damage and gastroduodenal complications, which are commonly seen in external radiotherapy [40]. In our study, no severe AE caused by irradiation occurred and ≥ grade 3 seed implantation-related AEs were only observed in two patients.

Our study had some limitations. First, this study was a retrospective study. Since iodine-125 seed brachytherapy is a locoregional therapy that requires percutaneous transhepatic puncture, the patients with a good performance status, a preserved liver function and a smaller tumor burden were more likely to be recommended for combining this treatment. This treatment preference inevitably lead to selection bias. However, the bias was limited by applying a PSM analysis. Second, two TKIs were used in treatment of patients. Although subgroup analyses showed that the combination of iodine-125 brachytherapy could bring better survival benefits to the patients treated with either sorafenib or lenvatinib, the inconformity of treatment and its potential impact on clinical outcomes deserved attention. Third, the sample size of this study was limited. It is necessary to validate our findings with further large-scale randomized trials.

## Conclusions

Our study showed safety and promising outcomes with the combination treatment of TKI-I in TACE-refractory patients. These patients could benefit from TKI-I and had significantly better tumor responses and improved survival in comparison with TKI alone. With the support of these findings, a randomized phase III trial comparing lenvatinib plus iodine-125 seed brachytherapy and lenvatinib alone for TACE-refractory HCC is ongoing (ClinicalTrials.gov; identifier: NCT05608213).

### Electronic supplementary material

Below is the link to the electronic supplementary material.

Supplementary Material 1

Supplementary Material 2

## Data Availability

The datasets used and/or analysed during the current study are available from the corresponding author on reasonable request.

## Abbreviations

HCC: Hepatocellular carcinoma
TACE: Transarterial chemoembolization
TKI: Tyrosine-kinase inhibitor
TKI-I: Tyrosine-kinase inhibitor combined with iodine-125 seed brachytherapy
ECOG PS: Eastern Cooperative Oncology Group performance status
CT: Computed tomography
MRI: Magnetic resonance imaging
CR: Complete response
PR: Partial response
SD: Stable disease
PD: Progressive disease
ORR: Objective response rate
DCR: Disease control rate
OS: Overall survival
TTP: Time to progression
AE: Adverse event
TRAE: Treatment-related adverse event
PSM: Propensity score matching
CI: Confidence interval
HR: Hazard ratio
