# Supplementary material for: Tyrosine-kinase inhibitor combined with iodine-125 seed brachytherapy for hepatocellular carcinoma refractory to transarterial chemoembolization: a propensity-matched study
Source: Cancer Imaging. 2023 Sep 25;23:91. doi: 10.1186/s40644-023-00604-4 (PMC10518921; doi:10.1186/s40644-023-00604-4)
Supplement: Supplementary file 1 — Supplementary Material 1 [file 40644_2023_604_MOESM1_ESM.docx]

**Table S1** Analyses of prognostic factors for overall survival and time to progression in the matched cohort

| **Factor** | **Overall survival** | | | | |  | **Time to progression** | | | | |
| --- | --- | --- | --- | --- | --- | --- | --- | --- | --- | --- | --- |
|  | **Univariate analysis** | |  | **Multivariate analysis** | |  | **Univariate analysis** | |  | **Multivariate analysis** | |
|  | **HR (95% CI)** | ***P*** |  | **HR (95% CI)** | ***P*** |  | **HR (95% CI)** | ***P*** |  | **HR (95% CI)** | ***P*** |
| Treatment option |  |  |  |  |  |  |  |  |  |  |  |
| TKI/TKI-I | 3.465 (2.017-5.952) | <0.001 |  | 3.546 (2.015-6.239) | <0.001 |  | 3.343 (2.019-5.534) | <0.001 |  | 3.305 (1.965-5.558) | <0.001 |
| Sex |  |  |  |  |  |  |  |  |  |  |  |
| Male/Female | 1.337 (0.636-2.811) | 0.443 |  |  |  |  | 2.515 (1.148-5.506) | 0.021 |  |  | 0.182 |
| Age (years) |  |  |  |  |  |  |  |  |  |  |  |
| <60/≥60 | 1.137 (0.698-1.853) | 0.605 |  |  |  |  | 1.186 (0.768-1.831) | 0.441 |  |  |  |
| ECOG PS |  |  |  |  |  |  |  |  |  |  |  |
| 1/0 | 1.180 (0.696-2.003) | 0.539 |  |  |  |  | 0.898 (0.560-1.441) | 0.656 |  |  |  |
| HBsAg |  |  |  |  |  |  |  |  |  |  |  |
| Positive/Negative | 1.886 (0.812-4.376) | 0.140 |  |  |  |  | 1.510 (0.753-3.029) | 0.246 |  |  |  |
| Child-Pugh class |  |  |  |  |  |  |  |  |  |  |  |
| B/A | 1.315 (0.567-3.055) | 0.524 |  |  |  |  | 0.702 (0.304-1.622) | 0.407 |  |  |  |
| AFP (μg/L) |  |  |  |  |  |  |  |  |  |  |  |
| ≥200/<200 | 1.566 (0.956-2.564) | 0.075 |  | 1.722 (1.038-2.857) | 0.035 |  | 1.365 (0.884-2.107) | 0.160 |  |  |  |
| Number of tumors |  |  |  |  |  |  |  |  |  |  |  |
| >3/≤3 | 2.179 (1.313-3.615) | 0.003 |  | 1.981 (1.172-3.348) | 0.011 |  | 2.282 (1.403-3.712) | 0.001 |  |  | 0.515 |
| Tumor distribution |  |  |  |  |  |  |  |  |  |  |  |
| Bilobar/Unilobar | 1.808 (1.088-3.005) | 0.022 |  |  | 0.515 |  | 2.592 (1.572-4.273) | <0.001 |  | 2.513 (1.497-4.218) | <0.001 |
| Largest tumor size (cm) |  |  |  |  |  |  |  |  |  |  |  |
| >7.0/≤7.0 | 1.559 (0.918-2.649) | 0.101 |  |  |  |  | 1.881 (1.170-3.024) | 0.009 |  |  | 0.318 |
| Macrovascular invasion |  |  |  |  |  |  |  |  |  |  |  |
| Yes/No | 1.382 (0.751-2.545) | 0.299 |  |  |  |  | 1.299 (0.768-2.197) | 0.330 |  |  |  |
| TKI |  |  |  |  |  |  |  |  |  |  |  |
| Sorafenib/Lenvatinib | 1.643 (0.996-2.711) | 0.052 |  |  | 0.403 |  | 1.847 (1.162-2.935) | 0.009 |  | 2.037 (1.267-3.276) | 0.003 |
| Number of previous TACE |  |  |  |  |  |  |  |  |  |  |  |
| 2/>2 | 1.711 (1.039-2.818) | 0.035 |  | 1.938 (1.160-3.238) | 0.012 |  | 1.580 (1.008-2.475) | 0.046 |  | 1.801 (1.139-2.846) | 0.012 |
| TACE technique |  |  |  |  |  |  |  |  |  |  |  |
| D-TACE/cTACE | 1.194 (0.733-1.944) | 0.476 |  |  |  |  | 1.623 (1.037-2.540) | 0.034 |  |  | 0.558 |

Analyses were performed using Cox proportional hazard regression model. *HR* hazard ratio, *CI* confidence interval, *TKI* tyrosine kinase inhibitor, *TKI-I* tyrosine kinase inhibitor combined with iodine-125 seed brachytherapy, *ECOG PS* Eastern Cooperative Oncology Group Performance Status, *HBsAg* hepatitis B surface antigen, *AFP* α-fetoprotein, *TACE* transarterial chemoembolization, *D-TACE* drug-eluting bead transarterial chemoembolization, *cTACE* conventional transarterial chemoembolization

**Table S2** Tumor responses for the patients in total cohort

| **Response** | **Overall tumor** | | |  | **Intrahepatic tumor** | | |  | **Vascular tumor thrombus** | | |
| --- | --- | --- | --- | --- | --- | --- | --- | --- | --- | --- | --- |
|  | **TKI-I group**  **(*n*=68)** | **TKI group**  **(*n*=64)** | ***P*** |  | **TKI-I group**  **(*n*=68)** | **TKI group**  **(*n*=64)** | ***P*** |  | **TKI-I group**  **(*n*=55)** | **TKI group**  **(*n*=48)** | ***P*** |
| CR, *n* (%) | 3 (4.4) | 1 (1.6) |  |  | 4 (5.9) | 1 (1.6) |  |  | 20 (36.4) | 0 (0.0) |  |
| PR, *n* (%) | 39 (57.4) | 17 (26.6) |  |  | 39 (57.4) | 18 (28.1) |  |  | 21 (38.2) | 6 (12.5) |  |
| SD, *n* (%) | 18 (26.5) | 24 (37.5) |  |  | 19 (27.9) | 25 (39.1) |  |  | 10 (18.2) | 25 (52.1) |  |
| PD, *n* (%) | 8 (11.8) | 22 (34.4) |  |  | 6 (8.8) | 20 (31.3) |  |  | 4 (7.3) | 17 (35.4) |  |
| ORR, % | 61.8 | 28.1 | <0.001 |  | 63.2 | 29.7 | <0.001 |  | 74.5 | 12.5 | <0.001 |
| DCR, % | 88.2 | 65.6 | 0.002 |  | 91.2 | 68.8 | 0.001 |  | 92.7 | 64.6 | <0.001 |

*TKI-I* tyrosine kinase inhibitor combined with iodine-125 seed brachytherapy, *TKI* tyrosine kinase inhibitor, *CR* complete response, *PR* partial response, *SD* stable disease, *PD* progressive disease, *ORR* objective response rate, *DCR* disease control rate

**Table S3** Treatment-related adverse events in the total cohort

| **Adverse events** | **Any grade** | | |  | **≥Grade 3** | | |
| --- | --- | --- | --- | --- | --- | --- | --- |
|  | **TKI-I group**  **(*n* = 68)** | **TKI group**  **(*n* = 64)** | ***P*** |  | **TKI-I group**  **(*n* = 68)** | **TKI group**  **(*n* = 64)** | ***P*** |
| Total | 61 (89.7) | 59 (92.2) | 0.620 |  | 23 (33.8) | 21 (32.8) | 0.902 |
| Related to TKI | 61 (89.7) | 59 (92.2) | 0.620 |  | 22 (32.4) | 21 (32.8) | 0.955 |
| Diarrhea | 25 (36.8) | 26 (40.6) | 0.649 |  | 4 (5.9) | 3 (4.7) | >0.999 |
| Hypertension | 24 (35.3) | 24 (37.5) | 0.792 |  | 13 (19.1) | 14 (21.9) | 0.695 |
| Hand-foot syndrome | 23 (33.8) | 19 (29.7) | 0.610 |  | 3 (4.4) | 4 (6.3) | 0.934 |
| Weight loss | 21 (30.9) | 18 (28.1) | 0.729 |  | 2 (2.9) | 1 (1.6) | >0.999 |
| Decreased appetite | 19 (27.9) | 20 (31.3) | 0.677 |  | 2 (2.9) | 2 (3.1) | >0.999 |
| Fatigue | 17 (25.0) | 14 (21.9) | 0.672 |  | 1 (1.5) | 0 (0.0) | >0.999 |
| Nausea | 12 (17.6) | 14 (21.9) | 0.542 |  | 1 (1.5) | 1 (1.6) | >0.999 |
| Proteinuria | 12 (17.6) | 13 (20.3) | 0.696 |  | 1 (1.5) | 2 (3.1) | 0.958 |
| Abdominal pain | 12 (17.6) | 9 (14.1) | 0.574 |  | 1 (1.5) | 1 (1.6) | >0.999 |
| Dysphonia | 12 (17.6) | 9 (14.1) | 0.574 |  | 0 (0.0) | 0 (0.0) | … |
| Arthralgia/myalgia | 11 (16.2) | 14 (21.9) | 0.404 |  | 0 (0.0) | 1 (1.6) | 0.976 |
| Elevated AST | 11 (16.2) | 9 (14.1) | 0.735 |  | 1 (1.5) | 0 (0.0) | >0.999 |
| Elevated ALT | 11 (16.2) | 8 (12.5) | 0.548 |  | 2 (2.9) | 0 (0.0) | 0.503 |
| Decreased platelet count | 10 (14.7) | 8 (12.5) | 0.712 |  | 2 (2.9) | 0 (0.0) | 0.503 |
| Rash | 10 (14.7) | 8 (12.5) | 0.712 |  | 0 (0.0) | 0 (0.0) | … |
| Vomiting | 9 (13.2) | 10 (15.6) | 0.696 |  | 1 (1.5) | 1 (1.6) | >0.999 |
| Decreased leukocyte count | 9 (13.2) | 9 (14.1) | 0.890 |  | 1 (1.5) | 0 (0.0) | >0.999 |
| Alopecia | 9 (13.3) | 8 (12.5) | 0.900 |  | 0 (0.0) | 0 (0.0) | … |
| Elevated TBi | 9 (13.3) | 7 (10.9) | 0.686 |  | 1 (1.5) | 0 (0.0) | >0.999 |
| Hypoalbuminemia | 8 (11.8) | 10 (15.6) | 0.518 |  | 0 (0.0) | 1 (1.6) | 0.976 |
| Decreased hemoglobin | 8 (11.8) | 9 (14.1) | 0.694 |  | 0 (0.0) | 0 (0.0) | … |
| Elevated ALP | 8 (11.8) | 7 (10.9) | 0.881 |  | 0 (0.0) | 0 (0.0) | … |
| Elevated GGT | 8 (11.8) | 5 (7.8) | 0.446 |  | 0 (0.0) | 0 (0.0) | … |
| Edema | 7 (10.3) | 9 (14.1) | 0.507 |  | 0 (0.0) | 1 (1.6) | 0.976 |
| Oral mucositis | 7 (10.3) | 8 (12.5) | 0.690 |  | 0 (0.0) | 0 (0.0) | … |
| Constipation | 7 (10.3) | 7 (10.9) | 0.905 |  | 0 (0.0) | 0 (0.0) | … |
| Hypothyroidism | 6 (8.8) | 10 (10.9) | 0.684 |  | 0 (0.0) | 0 (0.0) | … |
| Related to iodine-125 seed implantation | 10 (14.7) | … | … |  | 2 (2.9) | … | … |
| Bile duct dilation | 2 (2.9) | … | … |  | 0 (0.0) | … | … |
| Seed migration | 2 (2.9) | … | … |  | 0 (0.0) | … | … |
| Pneumothorax | 2 (2.9) | … | … |  | 0 (0.0) | … | … |
| Arterioportal fistula | 2 (2.9) | … | … |  | 0 (0.0) | … | … |
| Hemothorax | 1 (1.5) | … | … |  | 1 (1.5) | … | … |
| Track seeding | 1 (1.5) | … | … |  | 1 (1.5) | … | … |
| Subcutaneous hematoma | 1 (1.5) | … | … |  | 0 (0.0) | … | … |

Data were presented as *n* (%). *TKI-I* tyrosine kinase inhibitor combined with iodine-125 seed brachytherapy, *TKI* tyrosine kinase inhibitor, *AST* aspartate aminotransferase, *ALT* alanine aminotransferase, *TBi* total bilirubin, *ALP* alkaline phosphatase, *GGT* γ-glutamyl transpeptidase
