# Supplementary material for: Tyrosine-kinase inhibitor combined with iodine-125 seed brachytherapy for hepatocellular carcinoma refractory to transarterial chemoembolization: a propensity-matched study
Source: Cancer Imaging. 2023 Sep 25;23:91. doi: 10.1186/s40644-023-00604-4 (PMC10518921; doi:10.1186/s40644-023-00604-4)
Supplement: Supplementary file 2 — Supplementary Material 2 [file 40644_2023_604_MOESM2_ESM.docx]

Fig. S1


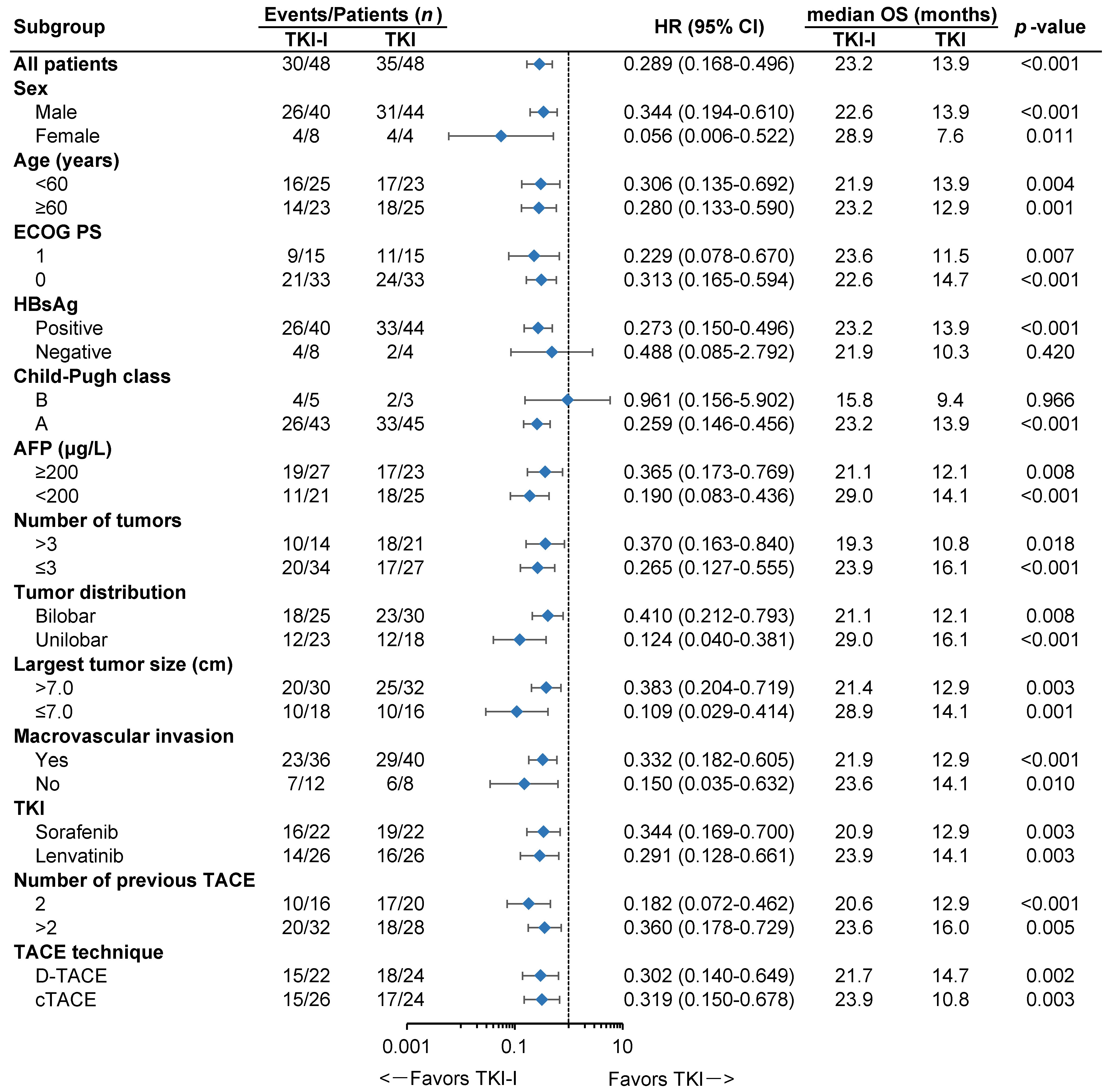


**Fig. S1** Forest plot of subgroup analyses for overall survival of the patients in matched cohort. *TKI-I* tyrosine-kinase inhibitor combined with iodine-125 seed brachytherapy, *TKI* tyrosine-kinase inhibitor, *HR* hazard ratio, *CI* confidence interval, *OS* overall survival, *ECOG PS* Eastern Cooperative Oncology Group Performance Status, *HBsAg* hepatitis B surface antigen, *AFP* α-fetoprotein, *TACE* transarterial chemoembolization, *D-TACE* drug-eluting bead transarterial chemoembolization, *cTACE* conventional transarterial chemoembolization
